# Effects of affective arousal on choice behavior, reward prediction errors, and feedback-related negativities in human reward-based decision making

**DOI:** 10.3389/fpsyg.2015.00592

**Published:** 2015-05-18

**Authors:** Hong-Hsiang Liu, Ming H. Hsieh, Yung-Fong Hsu, Wen-Sung Lai

**Affiliations:** ^1^Department of Psychology, National Taiwan UniversityTaipei, Taiwan; ^2^Department of Psychiatry, National Taiwan University HospitalTaipei, Taiwan; ^3^Graduate Institute of Brain and Mind Sciences, National Taiwan UniversityTaipei, Taiwan; ^4^Neurobiology and Cognitive Science Center, National Taiwan UniversityTaipei, Taiwan

**Keywords:** reward-based decision making, affective arousal, emotional regulation, emotional faces, feedback-related negativity (FRN), reward-prediction error (RPE), reinforcement learning model

## Abstract

Emotional experience has a pervasive impact on choice behavior, yet the underlying mechanism remains unclear. Introducing facial-expression primes into a probabilistic learning task, we investigated how affective arousal regulates reward-related choice based on behavioral, model fitting, and feedback-related negativity (FRN) data. Sixty-six paid subjects were randomly assigned to the Neutral-Neutral (NN), Angry-Neutral (AN), and Happy-Neutral (HN) groups. A total of 960 trials were conducted. Subjects in each group were randomly exposed to half trials of the pre-determined emotional faces and another half of the neutral faces before choosing between two cards drawn from two decks with different assigned reward probabilities. Trial-by-trial data were fit with a standard reinforcement learning model using the Bayesian estimation approach. The temporal dynamics of brain activity were simultaneously recorded and analyzed using event-related potentials. Our analyses revealed that subjects in the NN group gained more reward values than those in the other two groups; they also exhibited comparatively differential estimated model-parameter values for reward prediction errors. Computing the difference wave of FRNs in reward vs. non-reward trials, we found that, compared to the NN group, subjects in the AN and HN groups had larger “General” FRNs (i.e., FRNs in no-reward trials minus FRNs in reward trials) and “Expected” FRNs (i.e., FRNs in expected reward-omission trials minus FRNs in expected reward-delivery trials), indicating an interruption in predicting reward. Further, both AN and HN groups appeared to be more sensitive to negative outcomes than the NN group. Collectively, our study suggests that affective arousal negatively regulates reward-related choice, probably through overweighting with negative feedback.

## Introduction

Our daily life is flooded with making decisions, such as having milk or decaf latte for the morning, driving through the main road or taking a shortcut to the office, and holding or selling the declining stocks in hand. That said, optimal decisions are not easy to make, especially under the impact of emotion and other social factors (Bechara et al., 2000; Schwarz, 2000; Bechara, 2004; Winkielman et al., 2007; Pessoa, 2008). In recent years, the impact of emotional experience on decision making has attracted interests from psychologists, economists, and neuroscientists; however, its behavioral consequence and underlying neural mechanism remain unclear. Among the many kinds of decision processes, reward-based decision making has been extensively studied in both humans and animals (Schultz et al., 1998; Bayer and Glimcher, 2005; Rutledge et al., 2009; Glimcher, 2011; Hämmerer et al., 2011; Chen et al., 2012). It has also been investigated under the reinforcement-learning framework (Sutton and Barto, 1998), in which the goal of optimal behavior of an organism is to maximize its reward through the minimization of the *reward prediction error* (RPE).

RPE represents the difference between the value of expected and actual outcomes, and has been suggested to drive the learning process and choice behavior. A positive RPE is generated when the outcome of an option is better than expected, and would increase the (future) expected value of that option. In contrast, a worse-than-expected outcome leads to a negative RPE, and would decrease the expected value. Further evidence has shown that RPE signaling is not only expressed by mid-brain dopaminergic neurons (Schultz et al., 1997; Bayer and Glimcher, 2005) but also is carried out by an extensive fronto-subcortical network, including the orbitofrontal cortex, ventromedial pre-frontal cortex, anterior cingulate cortex, striatum, and amygdala (Hare et al., 2008; Haber and Knutson, 2010; Glimcher, 2011; Shenhav et al., 2013). In complementary to these imaging works disclosing neural circuits engaged in the decision process, activities of event-related potentials (ERP) are considered to be especially suitable for the capture of covert mental operations and dynamic changes because of the high temporal resolution and electrophysiological nature. Recently, researchers further identified a component termed *feedback-related negativity* (FRN) as a potential electrophysiological signature for coding RPE signals during reinforcement learning (Holroyd and Coles, 2002; Holroyd et al., 2003, 2009; Yasuda et al., 2004; Warren et al., 2014).

FRN is the difference wave computed by subtracting activities following positive outcomes from those following negative outcomes, and is commonly obtained at the fronto-central sites (e.g., Fz, FCz, and Cz) around 200–400 ms after the onset of the outcome feedback (Miltner et al., 1997). A dominant theory, dubbed the *reinforcement-learning error-related negativity* (RL-ERN; Holroyd and Coles, 2002) claims that FRN reflects RPE signaling derived from the dopaminergic projection to the anterior cingulate cortex and neighboring areas (Amiez et al., 2005; Mars et al., 2005; Walsh and Anderson, 2012; Warren et al., 2014). Increased RPE signals would disinhibit the anterior cingulate cortex (ACC) activity and lead to a larger FRN, while decreased RPE signals would inhibit the ACC activity and the FRN. Convergent evidence also indicates that FRN is affected by psychiatric or long-term emotional traits (Hajcak and Simons, 2002; Hajcak et al., 2003; Yasuda et al., 2004). Using emotional pictures (Lang et al., 1999) as the affective primes in a flanker task, Wiswede et al. (2009) reported that preceding unpleasant pictures can result in an enhanced error-related negativity (i.e., a counterpart of FRN) compared with the trials with neutral or pleasant pictures. Altogether, the above evidence implies that FRN not only indicates the dynamic of RPE signaling, but also reflects the impact of regulatory factors on the decision process.

Given that emotional experience has a pervasive impact on our decision-making and its underlying mechanism remains unclear, it is of great interest to explore the issue through the measurement of FRN and the evaluation of RPE-driven behavior. In particular, using a mixed-design with both within- and between-subjects comparisons of the facial-expression primes in a probabilistic learning task, this study aimed to investigate how affective arousal regulates the reward-based choice behavior from the behavioral, model-fitting, and ERP perspectives. There were three groups in this study, including the Neutral-Neutral (NN) group, Angry-Neutral (AN) group, and Happy-Neutral (HN) group. The NN group served as the group-wise baseline control and the neutral condition in the AN and HN groups served as the within-subject baseline control. Three types of facial expressions (i.e., neutral, angry, and happy faces) from a culture-based facial-expression database (Chen et al., 2009, 2013) were selected to elicit affective arousal for each corresponding group. Specifically, angry and happy faces were adopted as the affective primes and neutral faces were used as the neutral prime. Each subject in the three groups was confronted with either an affective-prime condition or a neutral-prime condition during each trial. We expect to observe an up-regulation for both the model-estimated and electrophysiological indices of RPE signaling by affective arousal for both the within- and between-subjects comparisons. Specifically, FRNs and the learning rate—a model-estimated parameter which is assumed to be positively correlated with RPE signaling, would be higher under the affective condition in the two emotional groups (i.e., AN and HN). On the contrary, the choice perseveration (consistency) parameter from the reinforcement learning model would be lower for the same case, as it is assumed to be negatively correlated with RPE signaling. Furthermore, this regulatory effect would also be manifested as unstable choices and thus interrupt performances in the behavioral level.

## Materials and methods

### Subjects

Sixty-six paid subjects recruited from National Taiwan University were randomly assigned to three groups: the NN group (8 males and 14 females; mean age: 21.14 years), the AN group (12 males and 10 females; mean age: 22.14 years), and the HN group (11 males and 11 females; mean age: 21.55 years). All subjects had normal or corrected-to-normal vision and were screened for the presence of psychiatric or neurological disorders. Informed consent was provided by each subject prior to the experiment in accordance with the procedure of the Research Ethics Committee of National Taiwan University.

### Design

**Figure 1:**
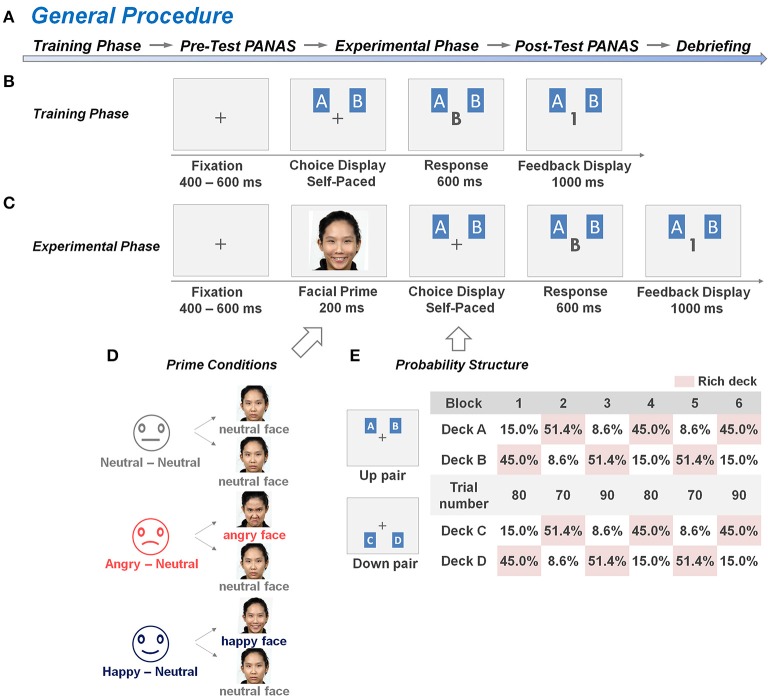
**General procedure and experimental design of the dynamic reward task. (A)** The general procedure sequentially consisted of a training phase, a pre-test PANAS rating phase, an experimental phase, a post-test PANAS rating phase, and a task debriefing phase. **(B,C)** An example of the trial structure in the training and experimental phases of the dynamic reward task, respectively. The training phase comprised a minimum of 40 training trials without a facial prime prior to each trial, whereas the experimental phase comprised 960 test trials with a facial prime prior to each trial. In each trial, the subjects had to choose one card from the two decks (either from deck pairs A, B or C, D) on the screen, and their choice was displayed on the center of the screen. When a reward had been scheduled to their choice, a digit “1” was displayed on the center of the screen to represent a one-point gain. Otherwise, the displayed digit was “0” to represent a non-rewarded outcome. **(D)** In the experimental phase of the dynamic reward task, the subjects from each of the three groups [the Neutral-Neutral (NN) group, Angry-Neutral (AN) group, and Happy-Neutral (HN) group] were randomly exposed to half of the trials with a pre-determined affective prime condition and half of the trials with a neutral prime condition prior to making trail-by-trial two-choice decisions. A set of natural faces from 8 individuals in our culture-based facial-expression database was selected and used for the corresponding affective or neutral prime conditions. **(E)** An example of a block sequence and the underlying reward-probability structure. Reward ratios of the two deck-pairs (6:1, 3:1, 1:3, 1:6) varied from block to block (70–90 trials per block). The blocks were separated by un-signaled transitions in which the higher reward probability was shifted within each deck pair, but with the reward probability otherwise unpredictable.

As illustrated in Figure 1A, the entire test consisted of five main phases, which included the training phase of the dynamic reward task, the pre-test questionnaire, the experimental phase of the dynamic reward task, the post-test questionnaire, and the debriefing. The dynamic reward task was modified from Rutledge et al. (2009) and Li et al. (2014), which employed a trial-by-trial two-card scenario as illustrated in Figures 1B,C. In each trial, two cards, including one card from a “rich” deck and the second card from a “poor” deck, were presented side by side on the computer screen without showing the reward values until the subject made a choice between them. Next, feedback of 0 (no reward) or 1 (reward) point was presented to the subject on the center of the screen. The subject was instructed to maximize the total points, and a monetary reward was provided to him/her based on his/her total game scores at the end of the experiment. The ratio of reward probabilities between the two decks varied across 6 blocks, and changes between blocks were not signaled to the subjects. The details of the procedure are described in the following section.

#### Training phase

Subjects were seated comfortably in front of a computer monitor in an electromagnetically shielded chamber. Following the setup with electroencephalogram (EEG) recording device and the task instruction, subjects answered three questions to confirm that they had a basic understanding of the task (see Supplementary 1). They were then engaged in the dynamic reward task which is a modified version of the probabilistic learning task (Rutledge et al., 2009; Li et al., 2014). The training phase comprised a minimum of 40 trials without the insertion of the facial prime. As illustrated in Figure 1B, there were two pairs of decks (i.e., A, B and C, D) with the same reward probability ratio (6:1 or 1:6, a total of 60%), and each pair was assigned to one of the two prime conditions within each subject (Watanabe et al., 2013). The subjects were informed that each pair of decks would be presented randomly in half of the trials, and the reward probabilities were fixed throughout the block. In each trial, the subjects had to choose one card from the two decks on the screen, and then the central fixation on the screen would be replaced by the display of their choice (i.e., capital letter A/B or C/D). When a reward had been scheduled to their choice, a digit “1” was displayed on the center of the screen, which represented a one-point gain. Otherwise, the displayed digit was “0,” which represented a non-reward outcome. The subjects were asked to identify the rich deck (i.e., the deck with higher reward probability) of each pair of decks after they completed the trials, and the training phase would be repeated until their answers were correct.

The training phase was followed by the pre-test questionnaire phase as shown in Figure 1A. Each subject was asked to complete the Chinese version of the Positive and Negative Affect Schedule (PANAS) (Watson et al., 1988; Teng and Chang, 2006) as a measure of their baseline affective arousal before experiencing the facial primes in the experimental phase.

#### Experimental phase

The experimental phase of the dynamic reward task was conducted immediately after the completion of the pre-test PANAS. Each subject was informed that (1) the distribution of reward probabilities may shift over time; (2) each trial would be introduced with a task-irrelevant face picture prior to the choice display; and (3) his/her total game score in the experimental phase would be transmitted into an actual monetary reward in a 1:1 ratio. To investigate the emotional-regulation effect on reward-based decision making, a face picture was introduced prior to the presentation of the deck-pair to elicit affective arousal in each trial (Figure 1C). A split-plot design was adopted here. Subjects were randomly assigned into one of the three groups, and were exposed to one of the three types of facial expressions (i.e., happy, angry, and neutral faces) from the database (Chen et al., 2009, 2013) prior to choosing the card in each trial. Subjects in the AN (and HN, respectively) group were randomly exposed to angry (and happy, respectively) faces (as the affective prime) in half of the trials and to neutral faces (as the neutral prime) in the other half of the trials (Figure 1D). For the group-wise baseline control, subjects in the NN group were exposed to neutral faces in all the trials. Following Watanabe et al. (2013), we used two versions of displays of the deck-pair: A, B and C, D (Figure 1E); they were referred to as the *Up* and *Down* pairs. There were 480 trials in each prime condition (and so the total was 960 trials for each subject). These trials were divided into six 70–90 trial-blocks with four possible reward probability ratios within each deck-pair (6:1, 3:1, 1:3, and 1:6; see Figure 1E for an example). The blocks were separated by un-signaled transitions in which the higher reward probability was shifted within each deck pair, but with the reward probability otherwise unpredictable. There was no time limit for response in each trial, but subjects typically completed the entire task in approximately 50 min. Following the experimental phase, each subject were required to complete the post-test PANAS, and the difference between the pre- and post-test PANAS score was evaluated to assess his/her change in affective arousal.

#### Debriefing

Following the post-test PANAS phase, subjects were required to answer a task-debriefing questionnaire regarding (1) their decision style, (2) self-prediction of the game score, and (3) their recall and ratings of emotional intensity for the facial primes in the experimental phase (see Supplementary 2). Finally, the total game score was announced by the experimenters, and the subjects were paid accordingly.

### Reinforcement learning model-based analysis

To explore the process that underlies RPE-driven choice behavior, the trial-by-trial choice data from all conditions for each subject were fitted with a standard reinforcement learning model (for technical discussions, see Watkins and Dayan, 1992; Sutton and Barto, 1998). This model is composed of a sensory component, which represents how information is updated, and a decision component, which represents how a choice is made. For the updating component, we used a simplified *Q-learning* model to characterize the dynamic of RPE signaling during the task. Specifically, the expected value of the chosen deck [for example, *Q*_*A*_ (*t*) for deck A in trial *t*] was updated according to the following rule (see also Rutledge et al., 2009; Li et al., 2014):

(1) $$Q_{A}(t+1)=Q_{A}(t)+\alpha \delta (t)$$

(2) $$\delta (t)=R_{A}(t)-Q_{A}(t)$$

where δ(*t*) is the RPE that represents the discrepancy between the experienced and expected reward, and *R_A_(t)* represents the actual outcome from choosing deck A in trial *t* with a value of 1 for reward and 0 otherwise. The *learning rate* parameter, denoted by α in Equation (1), determines how rapidly the estimation of the expected value is updated. A higher learning rate indicates that the expected value is updated more frequently, and thus recent outcomes would have a greater impact on the expected value compared with less recent outcomes. For the choice component, similar to Rutledge et al. (2009) and Li et al. (2014), we assumed that the probability of choosing deck A, *P*_*A*_(*t* + 1), is determined by the Boltzmann exploration (Kaelbling et al., 1996), which is a logistic form that assigns a weight to each of the actions:

(3) $$P_{A}(t+1)=\frac{e^{\beta Q_{A}(t)}}{e^{\beta Q_{A}(t)}+e^{\beta Q_{B}(t)}}$$

The *choice perseveration* (consistency) parameter, denoted by β in Equation (3), refers to the tendency to make choice guided by expected reward values. Higher choice perseveration indicates that the deck with a higher expected value is more likely to be chosen, and the choice would be completely random when the value of the choice perseveration equals 0.

**Figure 2:**
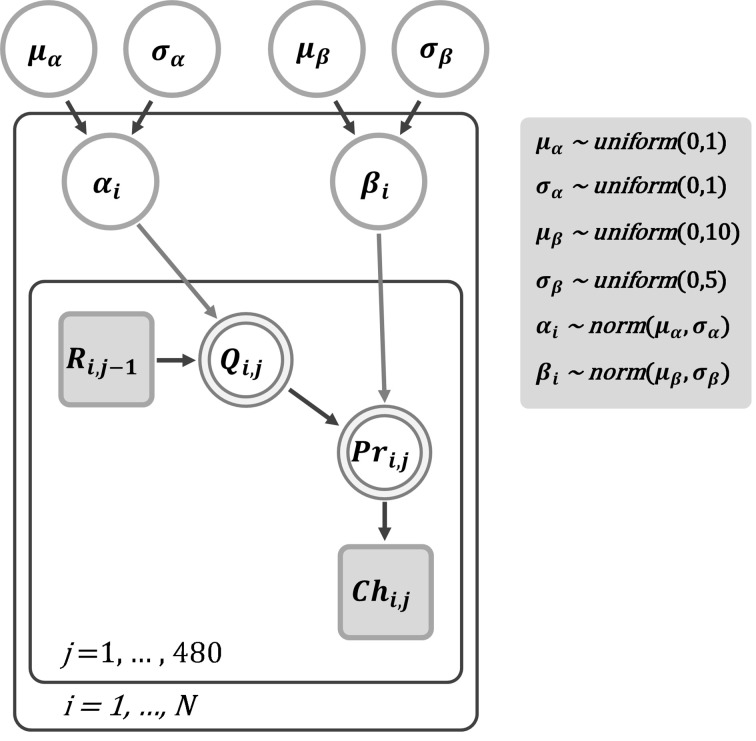
**A graphical Bayesian approach for parameter estimation of the reinforcement learning model for the dynamic reward task**. In this graphical model, nodes represent variables of interest and arrows indicate dependencies among these variables. Note that shaded nodes represent observed variables. Specifically, *R*_*i*, *j*−1_ is the reward received by subject *i* in trial *j* – 1, and *Ch_i, j_* is the observed choice of subject *i* in trial *j*. The parameters α_*i*_ and β_*i*_ represent the learning rate and choice perseveration for subject *i*, respectively. Each parameter was assumed from a normally distributed group-level population with respective means and standard deviations. In our implementation, both μ_α_ and σ_α_ were assigned an (non-informative) uniform distribution between 0 and 1 for the prior. For β, a uniform prior between 0 and 10 was assigned to μ_β_. For σ_β_, we assigned a uniform prior between 0 and 5.

To estimate the learning rate (α) and choice perseveration (β) in the reinforcement learning model, we applied a hierarchical Bayesian modeling approach with the Markov Chain Monte Carlo (MCMC) algorithm for parameter estimation to the trial-by-trial choice data from the dynamic reward task. The MCMC-based parameter estimation is commonly used in many fields, such as machine learning and computational psychology, and we have previously applied the same method to analyze the learning behavior of Akt1-mutant mice (Chen et al., 2012) and of patients with schizophrenia (Li et al., 2014). Such a method is especially suitable for dealing with multiple sources of variability within and between groups. The structure of the Bayesian hierarchical model we adopted is depicted in Figure 2. The parameters α and β for subject *i* (α_*i*_ and β_*i*_) were assumed normally distributed with respective means and standard deviations, which were from the group-level distributions (i.e., μ_α_ and σ_α_, and μ_β_ and σ_β_, respectively). We used WinBUGS (Lunn et al., 2000) to approximate the posterior distributions of the parameters by sampling values using the MCMC algorithm. Three MCMC chains were used for the estimation of α and β. A chain consisted of 16,000 iterations, of which the first 6000 (burn-in) points were discarded to ensure that only samples from the stationary distribution were used and that the data were unaffected by the starting values. Thus, we obtained 30,000 points of estimation from the three chains, from which we collected samples at intervals of every five samples, yielding a total of 6000 points. All interpretations and tests of the parameters were performed based on these 6000 samples. To ensure that the final estimation of posterior distributions would not be affected significantly by different priors, both μ_α_ and σ_α_ were assigned an (non-informative) uniform distribution between 0 and 1 for the prior. For β, a uniform prior between 0 and 10 and a uniform prior between 0 and 5 were assigned to μ_β_ and σ_β_, respectively.

### EEG recordings and data analysis

EEG signals were recorded with an electrode cap (Quick-Cap, NeuroScan, El Paso, Texas, USA) from 64 scalp locations according to the 10–10 system, using a SymAmp II amplifier (NeuroScan, El Paso, Texas, USA). The ground was placed above the forehead, and an electrode mounted in the middle position between Cz and CPz served as the online reference. The vertical and horizontal electrooculograms were recorded from the electrodes placed above and below the left eye and on the outer canthi of the left and right eyes, respectively. Impedances were kept below 5 kΩ. The sampling rate was 1 kHz with an online bandpass filter of 0.01–100 Hz. The recorded data were offline re-referenced to the average of the left and right mastoids using the EDIT module from Scan 4.5 (NeuroScan, Charlotte, North Carolina, USA) and then subjected to a 0.1–40 Hz bandpass filter and an EOG artifact reduction procedure, by which the continuous data were mathematically corrected for eye-blink artifacts through a built-in pattern recognition algorithm. The corrected continuous data were then segmented into epochs of −100 to 500 ms following the onset of the face picture to extract the ERP components for emotional and perceptual facial processing [i.e., early emotional positivity (EEP) and N170, respectively] (Bentin et al., 1996; Bentin and Deouell, 2000; Eimer, 2000; Eimer and Holmes, 2002, 2007; Eimer et al., 2003; Yovel et al., 2003; Sadeh et al., 2008), and epochs of −100 to 700 ms following the onset of feedback to extract ERPs for reward processing (i.e., FRN and its variation) (Holroyd and Coles, 2002; Holroyd et al., 2003, 2009; Yasuda et al., 2004). Baseline corrections were applied to the epoched data with respect to the mean activity of the pre-stimulus window. The epochs were then underwent an artifact rejection procedure in which the epochs that contained activities exceeding ±50 μV were excluded from further analysis, and the resulting average rejection rate was 6.19% across all conditions (NN group: 6.89%, AN group: 5.79%, HN group: 5.89%). The ERPs were obtained by averaging all the artifact-free epochs for each electrode and condition. For statistical analysis, the amplitude of the N170 following the facial prime was evaluated as the negative peak within the 110–200 ms post-stimulus interval at channels P7 and P8; the EEP amplitude was evaluated as the positive peak within the 120–180 ms post-stimulus interval at channels Fz, Cz, and Pz (Eimer and Holmes, 2002, 2007; Kiss and Eimer, 2008).

FRN is considered to reflect the processing of (negative) RPE signals in the brain (Holroyd and Coles, 2002; Holroyd et al., 2003, 2009; Yasuda et al., 2004; Marco-Pallares et al., 2008; Warren et al., 2014). Following Holroyd et al. (2009), in the present study, we distinguished between three types of difference waves of FRNs by subtracting the raw FRNs in the reward trials from those in the corresponding non-reward trials in the same condition. First, to extract the activities associated with reward processing from other overlapping ERP components, a *General FRN* was created by subtracting raw FRNs in all *reward* trials from those in all *non-reward* trials. Second, to characterize the baseline activity in feedback processing, an *Expected FRN* was created by subtracting raw FRNs in the *expected reward-delivery* trials (in which subjects chose cards from the rich deck and got the reward) from those in the *expected reward-omission* trials (in which subjects chose cards from the poor deck and didn't get the reward). Third, to characterize the activity related to expectation violation, an *Unexpected FRN* was created by subtracting raw FRNs in the *unexpected reward-delivery* trials (in which subjects chose cards from the poor deck but got the reward) from those in the *unexpected reward-omission* trials (in which subjects chose cards from the rich deck but didn't get the reward). Note that the Unexpected FRN has been considered to be an index of both positive and negative RPE signaling (Eppinger et al., 2008; Holroyd et al., 2009; Smillie et al., 2011; Cooper et al., 2014). For statistical analysis, amplitudes of these three difference waves were measured as the negative peak within the 200–400 ms post-stimulus interval^1^ and were evaluated at the three fronto-midline channels, Fz, FCz, and Cz, because the magnitude of FRNs is normally maximal at these sites (Holroyd and Krigolson, 2007; Holroyd et al., 2009).

### Statistical analyses

We computed multiple measures of subjective ratings, task performance, and ERP data. We also fit the trial-by-trial choice data with the standard reinforcement learning model. Statistical analyses were performed using SPSS 17.0 (SPSS Inc., Chicago, IL, USA) and SAS 8.1 for Windows (SAS Institute Inc., Cary, NC, USA). Repeated measures analysis of variance (RM-ANOVA) was used to assess behavioral and electrophysiological measures under the different combinations of the prime conditions. *Post-hoc* analyses were performed using Tukey's test when the *F*-value indicated a significant difference. A Greenhouse-Geisser adjustment of degrees of freedom, as well as a Bonferroni correction, was used when necessary. For the model-estimated parameters, instead of comparing the Bayes factors from each posterior distribution, Wald tests were applied to the estimated parameter values for a quick examination as previously described (Rutledge et al., 2009).

## Results

### Subjective ratings on affective arousal and task debriefing

**Figure 3:**
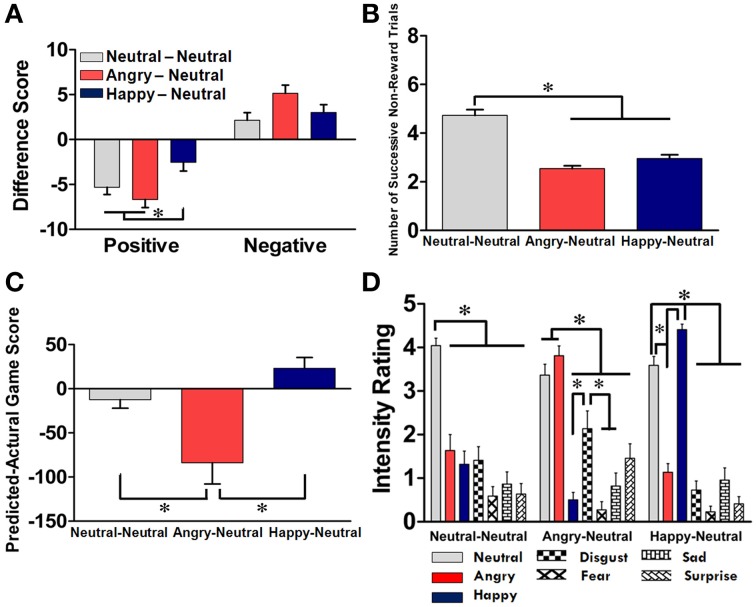
**Subjective ratings and task debriefing for the NN, AN, and HN groups. (A)** Differences between the post-test and pre-test PANAS rating scores on positive and negative affects across the three groups. **(B)** The numbers of successive non-reward trials prior to shifting. **(C)** Differences between self-prediction and actual game scores across the three groups. **(D)** Recall and intensity ratings of facial expressions in the experimental phase in each group. Data from the NN, AN, and HN groups are displayed in light gray, red, and deep blue bars, respectively, with the mean ± SEM and ^*^p < 0.05.

The effect of facial primes on the subjects' affective arousal was evaluated through the examination of the difference between the pre- and post-PANAS scores. As illustrated in Figure 3A, the mean difference on the rating scores across the three groups was −4.85 points for the positive affect and +3.42 points for the negative affect. *Post-hoc* analyses revealed that for the positive affect, the HN group showed less reduction than the other two groups (*p*s < 0.05); for the negative affect, no significant difference was identified among the three groups. A two-way RM-ANOVA revealed a significant main effect on the affect dimension [*F*_(1, 65)_ = 128.87, *p* < 0.001] and a significant interaction between affect dimension and subject group [*F*_(2, 126)_ = 6.49, *p* = 0.002]. Two separated one-way ANOVAs on each affect dimension further revealed that the effect of subject group was significant on the positive affect [*F*_(2, 65)_ = 5.52, *p* = 0.006], but was only marginal on the negative affect [*F*_(2, 65)_ = 3.02, *p* = 0.054]. As depicted in Figure 3B, we also found that the number of consecutive non-reward trials was significantly higher in the NN group compared with the other two groups [*F*_(2, 63)_ = 43.58, *p* < 0.001], suggesting that the subjects in the NN group tended to make more stable choices. Further evidence of the effect of affective priming was also supported from the analysis of the task-debriefing data. As shown in Figure 3C, a significant group effect was identified in the difference between the predicted and actual game scores [*F*_(2, 63)_ = 43.58, *p* < 0.001]. Subjects in the AN group underestimated their game scores compared with the other two groups (*p*s < 0.05); whereas subjects in the HN group tended to overestimate their performance, although the effect was marginal (*p* = 0.078). Furthermore, as shown in Figure 3D, different patterns on the recall and intensity ratings for the facial expression in the experimental phase were identified among the three groups. In each group, subjects' ratings for the emotion categories significantly corresponded with their assigned groups [*F*_(12, 440)_ = 18.36, *p* < 0.001]. These data support the success of our manipulation of affective priming in each group.

### Behavioral performance in the dynamic reward task

**Figure 4:**
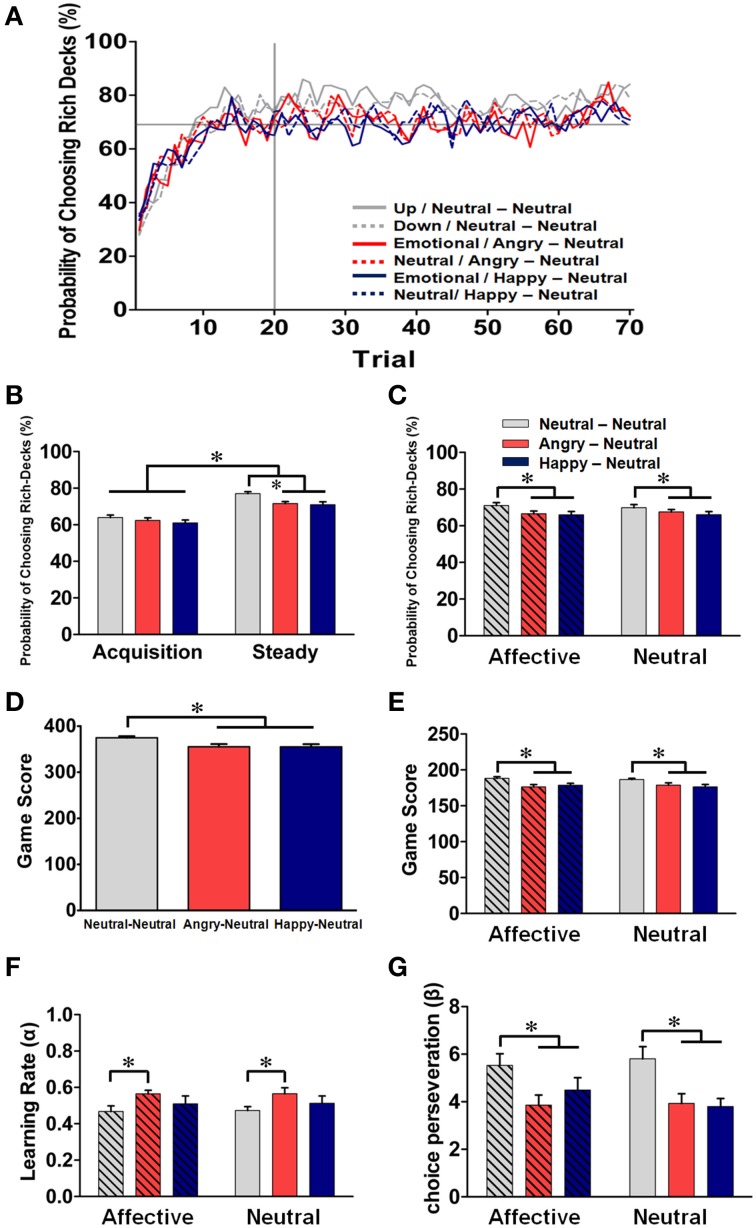
**Behavioral performance (mean ± SEM) in the dynamic reward task and parameter estimation in the model-based analysis across the three groups. (A)** Mean probabilities of choosing the rich-decks (PCRD) for the subjects in each of the three groups in both prime conditions. Each data point represented the moving average of 10 trials in each prime condition. The 1st–20th trials and the remaining trials in each block were defined as the “acquisition state” and the “steady state,” respectively. **(B)** Mean PCRD in the acquisition and steady states for the three groups. **(C)** Mean PCRD for each group under the affective and neutral prime conditions. **(D)** The overall total game scores for each group. **(E)** Mean game score for each group under the affective and neutral prime conditions. **(F)** Mean estimated learning rate for each group under the affective and neutral prime conditions. **(G)** Mean choice perseveration for each group under the affective and neutral prime conditions. For the line graphs, the groups are represented by different color lines (NN group: light gray; AN group: red; HN group: deep blue), and the two prime conditions in each subject group were distinguished by solid (affective) and dotted (neutral) lines. ^*^p < 0.05.

As depicted in Figure 4A, the mean *probability of choosing the rich-deck* (PCRD) was gradually accumulated across trials within each block, and it stabilized after the 20th trial. Therefore, the 1st–20th trials and the remaining trials in each block were considered as the *acquisition state* and the *steady state*, respectively. As shown in Figure 4B, the overall PCRD was significantly higher in the steady state (73.2%) than in the acquisition state (62.5%) (*p* < 0.001), reflecting the dynamic process of learning the distribution of reward probability and the formation of reward expectation. For the between-subjects comparison of the affective prime, no significant group effect was identified [*F*_(2, 63)_ = 0.67, *p* = 0.52] in the acquisition state; on the other hand, the NN group showed a significantly higher PCRD than the other two groups in the steady state [*F*_(2, 63)_ = 6.42, *p* = 0.002; *post-hoc* NN vs. AN: *p* = 0.017; NN vs. HN: *p* = 0.006].

Next, we turned to investigate the effect of affective prime-pairs on the behavioral performance of the three groups. As shown in Figure 4C, taking the whole trial-data for consideration, omnibus ANOVA revealed that there was a significant group effect for PCRD [*F*_(2, 63)_ = 3.66, *p* = 0.03], which suggesting that the NN group has a better PCRD than the other two groups in both prime conditions. However, neither the prime condition [*F*_(1, 65)_ = 0.002, *p* = 0.97] nor the interaction [*F*_(2, 63)_ = 0.82, *p* = 0.44] showed a significant difference for PCRD. Regarding the game score, as shown in Figure 4D, a significant group effect was found [*F*_(2, 63)_ = 8.28, *p* < 0.001] and the mean scores for the NN, AN, and HN groups were 374.86, 355.23, and 354.96, respectively. The NN group showed a higher game score than the other two emotional groups (both *p*s < 0.05). The mean game scores under the two prime conditions in each group are further depicted in Figure 4E. A two-way (prime condition × group) ANOVA revealed a significant main effect on group [*F*_(2, 63)_ = 5.02, *p* = 0.01]. *Post-hoc* analyses indicated that the NN group had a significantly higher game score than the other two emotional groups under both prime conditions (all *p*s < 0.05; Neutral vs. Angry: *p* = 0.003; Neutral vs. Happy: *p* = 0.002), and, once again, no significant effect was identified for the prime condition [*F*_(1, 63)_ = 0.15, *p* = 0.70] and for their interaction with group [*F*_(2, 63)_ = 1.28, *p* = 0.29]. Thus, our behavioral data indicated that the subjects' behavioral performance can be divided into two learning states in each block of the dynamic reward task, reflecting the dynamic of reward learning and expectation formation. More importantly, the NN group showed better PCRD and game scores than the other two emotional groups under both the affective and neutral prime conditions, suggesting a down regulation of behavioral performance by affective arousal.

### Model-based analysis and parameter estimation

To further investigate the process underlying the emotional regulation effect on subjects' performance in the dynamic reward task, a graphical Bayesian approach on the individual trial-by-trial choice data was applied to estimate the posterior distributions of the group mean (μ) and standard deviation (σ) for the learning rate (α) and choice perseveration (β) of the reinforcement learning model (see Equations 1–3). The Gelman–Rubin convergence statistics and a visual inspection indicated that the Markov chain converged properly. The estimated learning rate and choice perseveration for the three subject groups under the affective prime and neutral prime conditions are shown in Figures 4F,G, respectively. Wald tests indicate that the mean learning rate (μ_α_) was significantly higher in the AN group than in the NN group under both prime conditions (both *p*s < 0.005; corrected *p*s < 0.05; Figure 4F) and that the mean choice perseverations (μ_β_) was significantly higher in the NN group than in the other two emotional groups (*p*s < 0.005; corrected *p*s < 0.05; Figure 4G). The alteration of prime conditions within each group did not yield any significant effect. Though it is not entirely consistent with our behavioral data, different facial expressions did elicit differential effects on the parameters for RPE signaling, in which the NN group had a lower learning rate than the AN group and showed a higher degree of choice perseveration than both emotional groups. And, once again, the within-subject comparison of the prime condition showed no significant effect on the reward-driven choice behavior in each group.

### Event-related potentials (ERPs)

**Figure 5:**
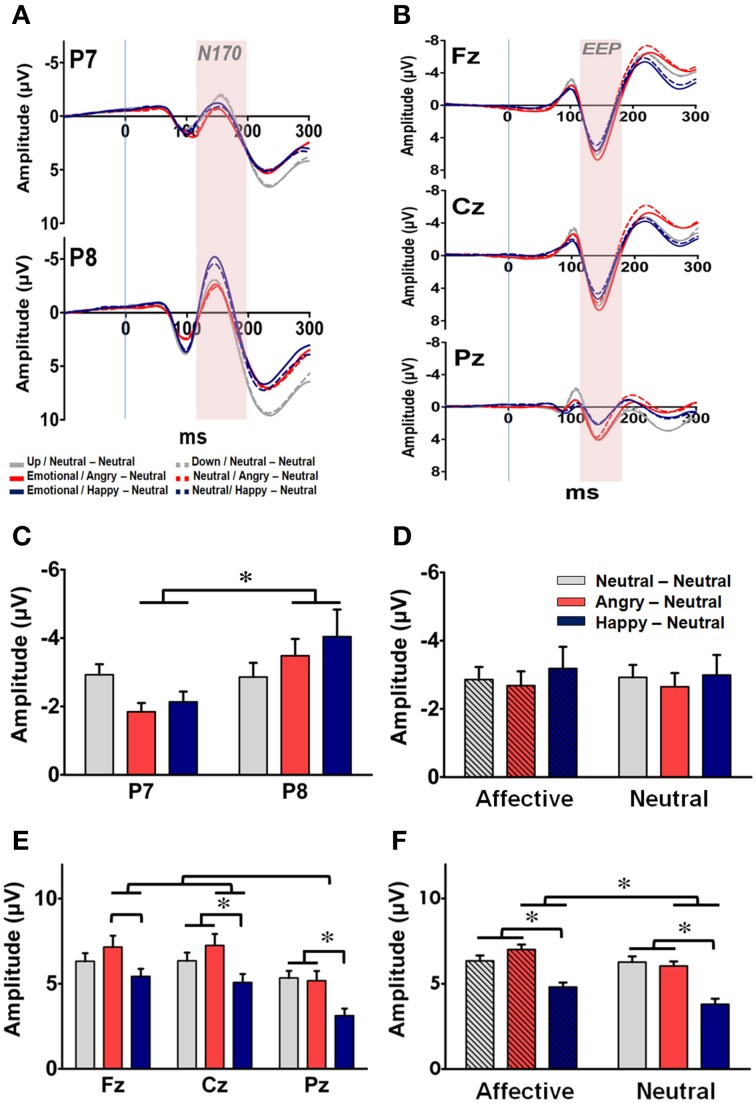
**Representative event-related potential waveforms and ERP components (mean ± SEM) for prime-processing across the three groups. (A)** Grand average of the ERP waveforms time-locked to the facial primes for each of the three groups at the two occipito-temporal sites (P7: upper panel; P8: lower panel). The time window of N170 is labeled by shaded color. **(B)** Grand average of the ERP waveforms time-locked to the facial primes for each of the three groups at the three midline sites (Fz: upper panel; Cz: middle panel; Pz: lower panel). The time window of the early emotional positivity (EEP) is labeled by shaded background. **(C)** Mean amplitude of N170 for each of the three groups at P7 and P8. **(D)** Mean amplitude of N170 for each of the three groups under the affective and neutral prime conditions. **(E)** Mean amplitude of EEP for each of the three groups at Fz, Cz, and Pz in each group. **(F)** Mean amplitude of EEP for each of the three groups under the affective and neutral prime conditions. ^*^p < 0.05.

Raw EEG data recorded during the experimental phase were processed into ERP components. The averaged ERP waveforms elicited by the facial primes under both prime conditions in each group at the bilateral occipito-temporal sites (i.e., P7 and P8) and the three midline sites (i.e., Fz, Cz, and Pz) are shown in Figures 5A,B, respectively. For the facial processing-related N170 recorded at P7 and P8 (Figure 5C), a three-way RM-ANOVA (electrode, prime condition, and group) revealed a significant main effect on electrode [*F*_(1, 63)_ = 6.55, *p* = 0.013] and a significant interaction between electrode and group [*F*_(2, 63)_ = 3.18, *p* = 0.048]. *Post-hoc* analyses indicated that the N170 was right-lateralized to the P8 electrode, especially in the AN and HN groups (*p*s < 0.05). No significant main effects were identified on either prime conditions [*F*_(1, 63)_ = 1.60, *p* = 0.21] or groups [*F*_(2, 63)_ = 1.60, *p* = 0.85] (Figure 5D). The finding of a constant right-lateralized N170 across different groups and prime conditions suggests that all facial primes were processed and recognized by the subjects.

For the EEP recorded at Fz, Cz, and Pz, a three-way RM-ANOVA (electrode, prime condition, and group) revealed significant main effects on electrode [*F*_(2, 126)_ = 25.48, *p* < 0.001], prime conditions [*F*_(1, 63)_ = 9.05, *p* = 0.004], and groups [*F*_(2, 63)_ = 11.45, *p* < 0.001]. The interactions between electrode × prime condition [*F*_(2, 126)_ = 7.14, *p* = 0.005], prime condition × group [*F*_(2, 63)_ = 14.94, *p* < 0.001], and electrode × prime condition × group [*F*_(4, 126)_ = 2.96, *p* = 0.042] were also significant. *Post-hoc* analyses indicated that the EEP was more prominent at the two fronto-central sites (i.e., Fz and Cz) than in the Pz electrode (*p*s < 0.001) (Figure 5E), and was larger under the affective prime condition than under the neutral prime condition for the two emotional groups (Figure 5F). Furthermore, as depicted in both Figures 5E,F, the amplitude and topographical distribution of EEP differ across both the prime conditions and subject groups. For subjects in the AN group, their mean EEPs elicited by the affective prime were larger than in the neutral prime condition at all three fronto-midline sites (*p*s < 0.05). A similar prime-condition effect was evident only at the Fz and Cz electrodes in the HN group (*p*s < 0.05), and was totally vanished in the NN group. Thus, the prime condition effect was stronger in the AN group, mild in the HN group, and totally vanished in the NN group. These findings suggest, as expected, that the manipulation of the facial prime elicited distinguished affective arousal in the two emotional groups.

**Figure 6:**
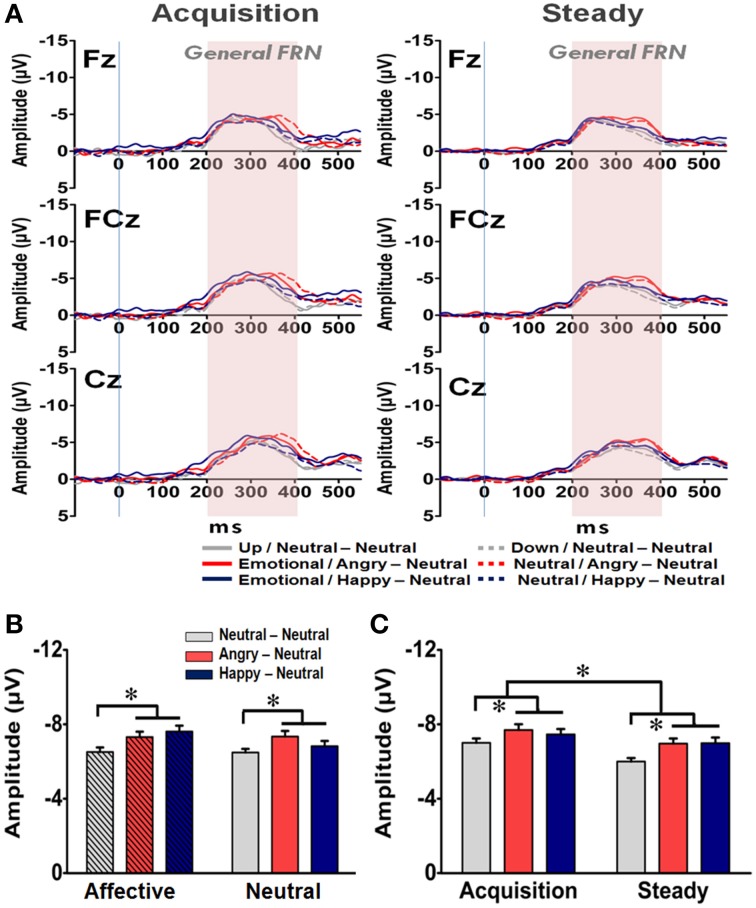
**Representative waveforms of the “General FRN” difference wave and their mean amplitudes (mean ± SEM) across the three groups. (A)** Grand averages of the difference wave time-locked to feedback display under the acquisition state (left panel) and steady state (right panel) in each subject group at the three fronto-midline electrodes. The time window of the General FRN is labeled by shaded background. **(B)** Mean amplitude in each subject group in the affective and neutral prime conditions. **(C)** Mean amplitude for each of the three groups in the acquisition and steady states. ^*^p < 0.05.

In addition to the N170 and EEP for the affective prime processing, the averaged waveforms for the General FRN at the Fz, FCz, and Cz electrodes are shown in Figure 6A. A four-way RM-ANOVA (electrode, prime condition, learning state, and group) revealed significant main effects on electrode [*F*_(2, 126)_ = 21.22, *p* < 0.001], learning state [*F*_(1, 65)_ = 11.74, *p* = 0.001], and group [*F*_(2, 63)_ = 5.57, *p* = 0.004]. *Post-hoc* analyses indicated that the mean amplitude of the General FRN was more pronounced at FCz (−7.22 μV) and Cz (−7.30 μV) than at Fz (−6.55 μV) (*p*s < 0.001) (Figure 6B), and it was higher in the acquisition state (−7.39 μV) than in the steady state (−6.65 μV) (Figure 6C). Given that the amount of prediction errors ought to decrease from the acquisition state to the steady state, it is very likely that the General FRN observed here might simply reflect the dynamic of RPE signaling in the brain as reported previously (Holroyd and Coles, 2002; Holroyd et al., 2003; Yasuda et al., 2004). More importantly, as shown in both Figures 6B,C, the mean amplitudes of the General FRN were higher in the two emotional groups (AN: −7.33 μV; HN: −7.23 μV) than in the NN group (−6.50 μV) and no significant effect was found on the prime condition [*F*_(1, 63)_ = 1.14, *p* = 0.29]. As the General FRN reflects the dynamic of RPEs, these findings suggest that both positive and negative arousal enhanced RPE signaling during the reward-based decision making.

**Figure 7:**
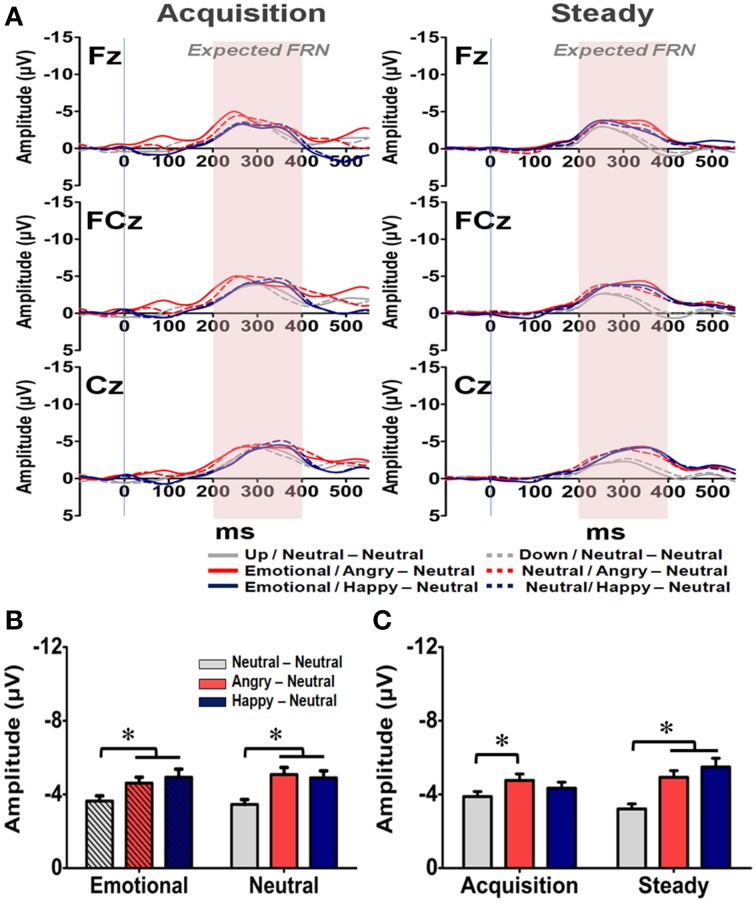
**Representative waveforms of the “Expected FRN” difference wave and their mean amplitudes (mean ± SEM) across the three groups. (A)** Grand averages of the difference wave time-locked to feedback display under the acquisition state (left panel) and steady state (right panel) in each subject group at the three fronto-midline electrodes. The time window of the Expected FRN is labeled by shaded background. **(B)** Mean amplitude in each subject group in the affective and neutral prime conditions. **(C)** Mean amplitude for each of the three groups in the acquisition and steady states. ^*^p < 0.05.

The averaged waveforms for the Expected FRN at Fz, FCz, and Cz electrodes are illustrated in Figure 7A. A four-way RM-ANOVA (electrode, prime condition, learning state, and group) revealed significant main effects on electrode [*F*_(2, 126)_ = 4.279, *p* = 0.033], learning state [*F*_(1, 65)_ = 5.257, *p* = 0.025], group [*F*_(2, 63)_ = 10.556, *p* < 0.001] and interactions between electrode × learning state [*F*_(2, 126)_ = 4.473, *p* = 0.023], electrode × group [*F*_(4, 126)_ = 3.21, *p* = 0.035], and electrode × learning state × group [*F*_(4, 126)_ = 6.877, *p* = 0.001]. As illustrated in Figure 7B, the mean amplitude was more pronounced at Cz than *Fz* (*p* = 0.001), which suggests a similar topographical distribution with the General FRN. *Post-hoc* analyses revealed that the two emotional groups exhibited larger Expected FRN than the NN group (*p*s < 0.05), and the difference of mean amplitude between the AN and NN groups was more pronounced than the one between the HN and NN groups (Figures 7B,C). Specifically, for the AN group, larger Expected FRNs than the NN group were evident at all three fronto-central sites across the acquisition and steady states (*p*s < 0.05). In contrast, compared with the Expected FRN in the NN group, the HN group exhibited a larger Expected FRN only at the Cz electrode in the acquisition state and at the FCz and Cz electrodes in the steady state (*p*s < 0.05). Although further studies are needed, one possible explanation is that the engagement of positive arousal in the HN group might enlarge neural population in the circuit representing the dynamic of RPE signaling. But no significant effect was found in the prime condition [*F*_(1, 63)_ = 1.19, *p* = 0.28] and its interactions with other factors. Thus, these results suggest that both positive and negative arousal enhanced RPE signals even when the reward outcome is expected, and the impact of negative arousal was even more pronounced than the positive one.

**Figure 8:**
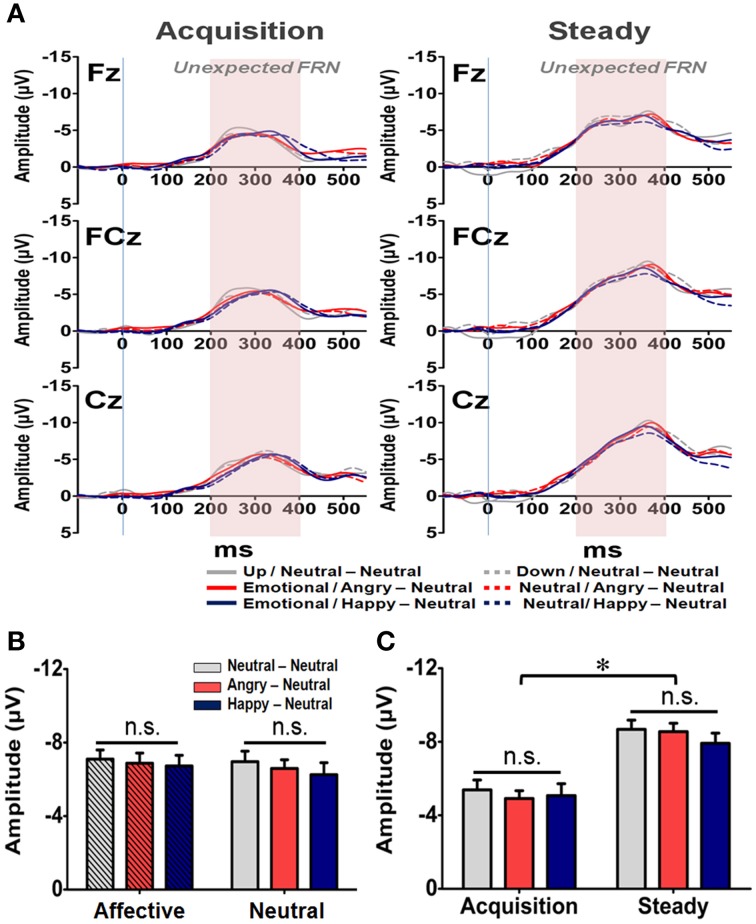
**Representative waveforms of the “Unexpected FRN” difference wave and their mean amplitudes (mean ± SEM) across the three groups**. **(A)** Grand averages of the difference wave time-locked to feedback display under the acquisition state (left panel) and steady state (right panel) in each subject group at the three fronto-midline electrodes. The time window of the Unexpected FRN is labeled by shaded background. **(B)** Mean amplitude in each subject group in the affective and neutral prime conditions. **(C)** Mean amplitude for each of the three groups in the acquisition and steady states. ^*^p < 0.05.

In addition to the Expected FRN, the averaged waveforms for the Unexpected FRN at the Fz, FCz, and Cz electrodes are illustrated in Figure 8A. A four-way RM-ANOVA (electrode, prime condition, learning state, and group) revealed significant main effects on electrode [*F*_(2, 126)_ = 35.79, *p* < 0.001] and learning state [*F*_(1, 65)_ = 24.10, *p* < 0.001]. *Post-hoc* analyses revealed that the topographical distribution of the Unexpected FRN across the three fronto-central electrodes was similar to those of the General and Expected FRNs. As shown in Figure 8B, the mean amplitude of the Unexpected FRN was more pronounced at FCz (−7.03 μV) and Cz (−7.48 μV) than Fz (−5.76 μV) (*p*s < 0.001). However, as depicted in Figure 8C, the Unexpected FRN was larger in the steady state (−8.34 μV) than in the acquisition state (−5.13 μV). Furthermore, no significant main effect or interaction was found in either prime condition [*F*_(1, 63)_ = 0.21, *p* = 0.65] or group [*F*_(1, 63)_ = 0.10, *p* = 0.91]. These results suggest that all subjects, regardless of their affective arousal, showed the same level of responses while confronting unexpected feedbacks.

## Discussion

In this study, an integrated approach was adopted to investigate how affective arousal regulates the decision making process in a probabilistic learning task. Our data indicate that (1) the inserted facial primes successfully altered subjects' affective arousal which was reflected in their PANAS ratings, task debriefings, and ERP components related to facial and affective processing (i.e., N170 and EEP); (2) subjects with affective arousal had lower PCRDs and game scores in the two emotional groups (i.e., AN and HN groups); (3) the between-subjects manipulation of affective arousal yielded a significant impact on reward processing—both the estimated model parameters and the General and Expected FRNs were enhanced in the two emotional groups; (4) the within-subject comparison of affective arousal showed no significant effect on RPE signaling in each group; (5) affective arousal rendered the subjects to be more sensitive to negative outcomes, which was reflected in larger Expected FRNs in the two emotional groups. To the best of our knowledge, this is the first study to integrate model-based analysis and ERP components to simultaneously examine the effects of affective primes with both positive and negative facial expressions on the regulation of reward-based decision making. Our results suggest that affective arousal primed by facial expressions have enduring effects across trials; it also negatively regulates reward-based decision making.

It has been well-accepted that emotions play a vital role in our decision making (Bechara et al., 2000; Schwarz, 2000; Bechara, 2004; Winkielman et al., 2007; Pessoa, 2008) even though the precise function remains debatable. In this study, a probabilistic dynamic reward task, which was modified from the “dynamic foraging task” for Parkinson's patients (Rutledge et al., 2009), the “dynamic reward task” for schizophrenic patients (Li et al., 2014), and the “dynamic foraging T-maze” for mice (Chen et al., 2012), was used to study how affective arousal regulates reward-based decision making, especially under uncertainty. To ensure and motivate our subjects in the performance of this task, actual monetary rewards were applied to mimic real-life events. Three types of facial expressions from the culture-based facial-expression database (Chen et al., 2009, 2013) were selected to prime affective arousal in each group. Patterns on the PANAS and facial-expression ratings evidenced that facial primes were perceived by our subjects and successfully elicited corresponding affective arousal as planned, and this was further confirmed by the N170 and EEP results.

It is not surprising to observe that subjects across groups, after participating in a 1-h ERP experiment, showed decreased positive affect and increased negative affect on their PANAS scores. Nevertheless, the AN group exhibited more negative arousal and underestimated their performance, whereas the HN group showed more positive arousal and tended to overestimate their game scores. As the N170 component is considered an index of online perceptual integration of facial features (Bentin et al., 1996; Bentin and Deouell, 2000; Eimer, 2000; Yovel et al., 2003), the typical right-lateralized N170 across all groups and conditions suggests that all facial primes used in this study were perceived by our subjects. The EEP component is assumed to reflect the early stage of affective processing (Eimer and Holmes, 2002, 2007; Kiss and Eimer, 2008), and thus the differences in amplitude and topographical distribution in each prime condition and subject group suggest that our manipulation was effective.

Moreover, affective arousal primed by facial expressions yielded significant impact on subjects' performance and, importantly, enhanced their RPE signaling during the decision process. It has been reported that the anticipation of viewing positive/rewarding pictures increased financial risk taking in a gambling task, and that the activation of the nucleus accumbens (which is the main dopaminergic target for reward) mediated the influence of reward cues on financial risk taking (Knutson and Bossaerts, 2007; Knutson et al., 2008; Heitland et al., 2012). In contrast, among most measurements we utilized here, the NN group consistently exhibited higher game scores and lower neural activities related to RPE signaling than the other two groups, whereas no significant difference was identified between the two emotional groups. Our reinforcement learning model-based analysis also revealed that the NN group had a higher degree of choice perseveration (consistency) compared with the other two emotional groups, which suggests that these control subjects had a higher tendency to make choices guided by reward values and their choices were more stable. Thus, the between-subjects manipulation of affective arousal was effective. In particular, exposure to angry or happy faces led to a similar negative effect on the regulation of choice behavior in the dynamic reward task. Accordingly, these findings appear to suggest that “stay calm” or “less emotional” might be a better strategy to gain additional rewards under uncertainty.

In addition to the emotional regulation obtained among the subject groups, we also examined whether the regulatory effect would sustain throughout the experimental task or could be transiently neutralized by the insertion of neutral facial primes. Using fearful and neutral facial primes, a recent fMRI study by Watanabe et al. (2013) provided a primal insight regarding probabilistic reward-based decision making. They found that the presentation of fearful faces as affective primes not only promoted subjects' behavioral performance, but also underscored the role of striatal-amygdala interactions in the modulation of RPE signaling (Watanabe et al., 2013). Further, they reported that the impact induced by the fearful faces can be ceased or neutralized by the presentation of neutral faces in the subsequent trials, which implies a transient effect on affective priming in decision making. In contrast, in our study, no significant difference was identified between the two prime conditions within each subject on the behavioral performance, estimated model-based parameters, and FRNs across subject groups. Our findings suggest that the presentation of emotional faces can evoke transient affective arousal, but its regulatory effect on reward-based decision making appears to be enduring rather than transient or rapidly switchable. In other words, although the within-subject manipulation of facial prime can alter subjects' affective arousal across testing trials, the emotional-regulation effect on decision making appears to be sustained across the entire task session. The inconsistent findings between our current study and Watanabe's study may result from the differences in the research purpose, design, and data analyses. Specifically, given that affective primes were presented before each choice-reward association in Watanabe's study, it is possible that subjects used affective primes to predict the identity of incoming choices and thus brought out stronger distinction between the two types of affective primes. On the other hand, in our current study, the two prime conditions were assigned to different deck-pairs with the same set of total reward probabilities, thus our subjects may tend to ignore the identities of the primes and treat the two deck-pairs as the same. This interpretation is supported by the fact that all subjects in this study claimed that they eventually adopted the same strategy on the two pairs of decks. Nevertheless, it is of great interest to further investigate the brain activity and neural circuits that underlie the emotional-regulation effect on reward-based decision making using neural imaging techniques and our probabilistic dynamic reward task.

Furthermore, our results of FRN difference waves in different conditions are consistent with our behavioral and model-fitting findings, which support the use of FRN as an index of RPE signaling (Holroyd and Coles, 2002; Yasuda et al., 2004; Holroyd and Krigolson, 2007). FRN has been shown to reflect the evaluation of monetary loss and negative performance feedback (Hajcak et al., 2007), and the modulation of the FRN has been suggested as a potential biomarker in psychopathology (Olvet and Hajcak, 2008). In the current study, three types of FRN were obtained and each of them was related to different aspects of reward processing. The General FRN is the difference wave derived by subtracting the raw FRN components in all rewarded trials from those in all non-reward trials. In this study, the mean amplitude of the General FRN was higher in the acquisition state than in the steady state in each task block. Given the fact that the amount of negative feedback decreased from the acquisition state to the steady state, the General FRN can be used as an index for the dynamic RPE signaling during reward-based decision making as previously reported (Holroyd and Coles, 2002; Holroyd et al., 2003, 2009; Yasuda et al., 2004; Marco-Pallares et al., 2008; Warren et al., 2014). It was also reported that this index is prone to overestimate the impact of negative outcomes (Holroyd and Coles, 2002; Holroyd et al., 2003, 2009; Yasuda et al., 2004; Marco-Pallares et al., 2008). Thus, in our current study, it is probable that the higher General FRN observed in the two emotional groups could be resulted from the enhancement of RPE signals induced by affective arousal. It also suggests that both the positive and negative components of emotional arousal can positively regulate RPE signaling during reward-based decision making. In addition to the General FRN, two additional types of FRN, namely the Expected FRN and Unexpected FRN, were conducted to characterize the activities in the expectation-matched and expectation-violated conditions in reward processing, respectively (Holroyd et al., 2009). A significant group difference was also revealed by the Expected FRN, in which the two emotional groups also exhibited higher mean amplitudes than the NN group. Even though the reward outcome is expected, subjects in the AN and HN groups exhibited larger responses to negative feedback compared with the subjects in NN group, suggesting that affective arousal can facilitate baseline brain activity and enhance the sensitivity to negative feedback in the emotional groups. In contrast, no group difference was identified by the Unexpected FRN in both the acquisition and steady states. Given that Unexpected FRN reflects the combination of both positive and negative RPE signals (Eppinger et al., 2008; Holroyd et al., 2009; Smillie et al., 2011; Cooper et al., 2014), it is likely that this duplicated activity has reached its ceiling of response. Thus, the emotional regulation effect cannot be manifested on this component. Note that this is just one explanation for the null effect, and may be limited by the design and scope of the present study. Future work on this issue would require a more flexible design for single-trial analysis on both EEG and model-fitting data to provide more dynamic and sensitive measurement of RPE signal (as demonstrated in Frank et al., 2015).

There is an increasing number of studies proposing that FRN codes prediction errors associated with motivational salience rather than motivational value (Alexander and Brown, 2011; Talmi et al., 2013; Hauser et al., 2014), challenging the classic view held by the RL-ERN theory (Holroyd and Coles, 2002). For example, Talmi et al. (2013) compared FRNs elicited by appetitive (i.e., monetary reward) and by aversive (i.e., electrical pain) outcomes. Specifically, the authors compared (1) FRNs following unexpected pain delivery and those following unexpected pain omission (positive RPE), and (2) FRNs following unexpected reward delivery and those following unexpected reward omission (negative RPE). They found that in both cases the difference-wave FRNs showed a negative deflection, and thus concluded that FRNs reflect the violation of outcome expectation but not the valence of outcome. However, the General, Expected, and Unexpected FRNs in our study were all identified as negative deflections. Although their mean amplitudes were different, it is obvious that unexpectedness was not the only factor behind the generation of FRN. Instead, our data are likely to be explained by the RL-ERN theory, as FRN reflects RPE signaling with different valences (i.e., positive or negative). Besides, it is still unclear whether aversive stimuli could be directly coded by the midbrain dopaminergic system as in the case of appetitive stimuli (Schultz et al., 1997; Delgado et al., 2000).

Altogether, an integrated study from behavioral, model-fitting, and ERP approaches was conducted here to investigate the emotional-regulation effect on reward-based decision making. These findings indicate that the presentation of facial expressions can prime affective arousal which then intensifies negative RPE signaling (especially for angry faces), dampens the sustainability for non-rewarded trials, interrupts reward expectation and eventually leads to the interference with gaining scores. Future research on the emotional-regulation effect of reward-based decision making would be timely and greatly worthwhile, especially using patients with abnormality in midbrain dopaminergic system (e.g., schizophrenia and pathological gambling).

### Conflict of interest statement

The authors declare that the research was conducted in the absence of any commercial or financial relationships that could be construed as a potential conflict of interest.

## Footnotes

^1^Note that the peak of difference wave (i.e., General FRN) is not necessarily the difference of the two respective peaks in non-reward and reward raw FRNs. Similar remarks apply to the Expected FRN and Unexpected FRN. Our use of the base-to-peak measure for difference-wave FRN follows closely the work by Holroyd and colleagues (Holroyd and Krigolson, 2007; Holroyd et al., 2003, 2009).
