# Supplementary material for: Effects of affective arousal on choice behavior, reward prediction errors, and feedback-related negativities in human reward-based decision making
Source: Front Psychol. 2015 May 18;6:592. doi: 10.3389/fpsyg.2015.00592 (PMC4434921; doi:10.3389/fpsyg.2015.00592)
Supplement: Supplementary file 1 [file DataSheet1.DOCX]

**Supplementary 1: Post-training questions**

1. Which deck (deck A or B) has a higher reward-probability?

2. Which deck (deck C or D) has a higher reward-probability?

3. Does repeatedly choosing deck A and then getting the points decrease its reward-probability?

4. Does repeatedly choosing deck C and then getting the points decrease its reward-probability?

**Supplementary 2: Post-experiment task-debriefing questionnaire**

1. How many consecutive non-rewarded trials can you stand before you switch to the other deck?

2. Following question #1, did you use different strategies for deck-pairs A/B and C/D? If you did, please describe your strategy in detail.

3. There were 960 trials in the experimental phase of the dynamic-rewarding task. How many points did you think you had gained?

4. Were you aware that there was a human face presented immediately prior to each trial? If yes, please continue to answer the following question.

5. Following question #4, what types of emotional expressions did you perceive from these faces? Please rate their intensity on the following emotion categories using the 5-point scale below (Multiple choices).

**□** Happy weak 1 2 3 4 5 strong

**□** Angry weak 1 2 3 4 5 strong

**□** Disgust weak 1 2 3 4 5 strong

**□** Fear weak 1 2 3 4 5 strong

**□** Sad weak 1 2 3 4 5 strong

**□** Surprise weak 1 2 3 4 5 strong

**□** Neutral (Peaceful) weak 1 2 3 4 5 strong
